# Donor and recipient risk factors for the development of primary graft dysfunction following lung transplantation

**DOI:** 10.3389/fimmu.2024.1341675

**Published:** 2024-02-06

**Authors:** J. Asher Jenkins, Ricardo Verdiner, Ashraf Omar, Juan Maria Farina, Renita Wilson, Jonathan D’Cunha, Pedro Augusto Reck Dos Santos

**Affiliations:** ^1^ Department of Cardiothoracic Surgery, Mayo Clinic Arizona, Phoenix, AZ, United States; ^2^ Department of Anesthesia and Perioperative Medicine, Mayo Clinic Arizona, Phoenix, AZ, United States; ^3^ Division of Pulmonology and Critical Care Medicine, Mayo Clinic Arizona, Phoenix, AZ, United States

**Keywords:** transplant, lung transplantation, primary graft dysfunction, end-stage lung disease, immunosuppression, organ preservation

## Abstract

Primary Graft Dysfunction (PGD) is a major cause of both short-term and long-term morbidity and mortality following lung transplantation. Various donor, recipient, and technical risk factors have been previously identified as being associated with the development of PGD. Here, we present a comprehensive review of the current literature as it pertains to PGD following lung transplantation, as well as discussing current strategies to mitigate PGD and future directions. We will pay special attention to recent advances in lung transplantation such as *ex-vivo* lung perfusion, thoracoabdominal normothermic regional perfusion, and up-to-date literature published in the interim since the 2016 ISHLT consensus statement on PGD and the COVID-19 pandemic.

## Introduction

Lung transplantation (LTx) is the current mainstay of care for patients with end-stage pulmonary disease. Per the United Network for Organ Sharing (UNOS), 2,692 lung transplants were performed in the United States in 2022. Despite the growing number of lung transplants being performed, pulmonary allografts remain the least durable solid organ out of kidney, liver, and heart (1). Primary graft dysfunction (PGD) is a major cause of pulmonary allograft dysfunction in the immediate perioperative period carrying an incidence of 10-30% of patients in which a mortality rate up to 40% can be observed (2, 3). Though there is no consensus on the exact pathophysiology behind the development of PGD, ischemia-reperfusion injury is generally considered as a major contributor to its development for several reasons (4–6). Firstly, the lung is a unique organ with a dual blood supply; that of the pulmonary vasculature, and that of the bronchial vessels. During transplantation, the bronchial blood supply is disrupted, leaving distal airways more prone to ischemic injury. Secondly, reactive oxygen species generated by an influx of cytosolic calcium during warm and cold ischemic times in response to ATP generation creates a cycle of inflammatory changes and eventual endothelial damage, which leads to more inflammation from increased capillary permeability and cytokine release, ultimately leading to cell death in the donor allograft (6, 7). Furthermore, the milieu of donor and recipient risk factors, which we will discuss in this article, are also key in the pathophysiology of PGD (8, 9). Importantly, PGD is strongly associated with the development of chronic lung allograft dysfunction (CLAD), which is the primary cause of long-term morbidity and mortality after LTx (10, 11). Thus, it is critical to identify patients who are at risk of developing PGD and maximize the lifespan of the transplanted lung. In this review, we will discuss the current literature pertaining to the definition of PGD as well as donor and recipient factors which may contribute to the development of PGD.

## PGD: current definition

PGD is a consensus-based, standardized definition and grading system defined by the International Society for Heart and Lung Transplantation (ISHLT) (12, 13). PGD is clinically characterized by the presence of diffuse alveolar infiltrates on radiographic imaging and the severity of hypoxemia based on the PaO2 to FIO2 (P/F) ratio (**Table 1**). The diagnosis of PGD is that of exclusion; it can only be made after rejection, volume overload, pulmonary embolism, infectious, or structural mechanisms of graft dysfunction have been ruled out. Additional caveats of the grading system also specify Grade 3 PGD (PGD3) as patients who received mechanical circulatory support post-transplant as well as patients that required inhaled nitric oxide >48 hours following LTx. PGD is graded in the immediate postoperative period every 24 hours from time 0 to 72 hours post reperfusion. The guidelines reflect findings in the literature that PGD3 is associated with higher rates of postoperative bronchiolitis obliterans (BOS) in addition to both short and long-term mortality (14, 15). PGD3 carries a much worse prognosis than PGD1 or PGD2 with 5-year mortality rates as high as 60% for PGD3 and 30% for PGD1/2 (3). Importantly, PGD at 72 hours seems to be the most reliable prognostic indicator during the 0-to-72-hour post reperfusion assessment interval (3). The most recent 2016 ISHLT PGD criteria has received recent criticism, as different trends in outcomes for each PGD classification have been observed following its implementation. A 2020 retrospective analysis demonstrated a higher proportion of lower PGD grades than prior to 2016, however, short-term mortality of patients with PGD3 did not differ from the previous classification (16).

**Table 1:** Grading of PGD as per 2016 ISHLT guidelines.

PGD Grade	Mechanical Circulatory Support	Interstitial Infiltrates on Chest X-Ray	PaO2/FIO2 Ratio
0	No	No	>300
1	No	Yes	>300
2	No	Yes	200-300
3	Yes*	Yes	<200

*Requirement of Mechanical Circulatory Support is automatically graded as PGD3.

## Donor risk factors for development of PGD

### Extended criteria donors

The ideal lung donor criteria are defined as age between 20 and 55 years, P/F ratio >350 mmHg, no smoking history, normal chest x-ray, <5 days of mechanical ventilation, BAL with negative gram stain and bronchoscopy, and <4-6 hours of ischemic time (17, 18). However, these strict criteria have significantly limited the pool of available donors. Thus, many centers have advocated for use of extended donor criteria. The use of marginal donors has been extensively studied for other organs. For lungs, several studies suggest comparable outcomes to optimal donors (19–21). Below, we will discuss some of the risk factors within these extended criteria and their associations with PGD.

### Age

Early studies have implicated donor age >45 years or <21 years as being correlated with development of PGD (22, 23). More recent studies have failed to demonstrate a significant association between donor age and development of PGD; however, long-term outcomes tended to correlate negatively with donor age as well as a higher incidence of CLAD (24–28). In terms of extremes of age, recent literature has examined outcomes after using lungs from donors >70 years of age. These studies report no significant difference in incidence of PGD3 relative to donors aged less than 65 years (26, 29, 30). These findings may suggest that previous effects of donor age on outcomes may be mitigated with advances in perioperative care, organ preservation strategies, and by placing greater emphasis on biological rather than chronological age of the potential donor (31).

### Lung donation following trauma

Use of pulmonary allografts following sustained chest trauma has been controversial. However, this topic has garnered recent attention to minimize loss of allografts from the donor pool. A 2022 study examined this in detail, which demonstrated no difference in PGD3 at 72 hours, duration of ventilation, and long-term graft survival in patients who received allografts with pulmonary contusions compared to those who did not (32, 33). Further research is needed in this area to examine whether the extent of contusion serves as an effect modifier and what subset of contused lungs can safely be used to expand the donor pool. In addition to sustaining lung contusions, trauma patients frequently require transfusion of blood products during initial resuscitation. Transfusion of blood products has been identified as an independent risk factor for the development of PGD and serves as an important consideration when evaluating a potential donor (34).

Another uncommon factor to consider in accepting lung allografts from a polytrauma donor is the occurrence of donor-acquired fat embolism syndrome (DAFES). Description of DAFES is sparsely described in the literature and limited to a handful of case series. According to recent literature, 8 cases of DAFES have been reported, and 3 of which were discharged alive (35–37). In these case reports, each met criteria for PGD3 postoperatively, and each patient that survived developed CLAD. A salient point made by Jacob and colleagues is that it is unknown what percent of PGD are actually DAFES, as signs/symptoms of DAFES often mimic PGD (37). In addition, DAFES was only diagnosed post-mortem during autopsy in nearly every previously reported case, emphasizing that the actual incidence of DAFES is likely underappreciated. Thus, special attention must be given to identify fat embolism in polytrauma donors during retrograde flushing and bronchoscopy to mitigate PGD.

### Sex mismatch

Mismatch between donor and recipient sex has previously been implicated as a risk factor for developing PGD and adverse outcomes in kidney, liver, and heart transplantation (38–41). The data as it pertains to sex mismatch and PGD in LTx have been mixed. Studies from France, the UK, and Canada have reported significantly worse overall survival for female-to-male transplants, as well as an increased likelihood of PGD development (38, 42, 43). A 2013 single center analysis did not demonstrate any significant difference in sex mismatched LTx in terms of overall outcome and development of PGD (44). Given the above heterogeneity, paucity of investigation, and lack of recent data on this topic, further investigation is needed to elucidate the impact, if any, sex mismatch has on PGD and overall outcomes.

### Substance use

Other social/demographic donor characteristics which have been demonstrated to correlate with PGD are smoking and alcohol use (45–47). The 2016 ISHLT consensus guidelines go on to list donor smoking as a “definite” risk factor for developing PGD (13). However, a recent 2023 study indicated that donor smoking, even >20 pack years, was not significantly associated with the development of PGD (48). The study does not suggest that donor smoking is benign, as it also demonstrates slightly worse long-term outcomes, most pronounced at 5-years, among those that received lungs from a smoking donor (48). In addition, in risk-prediction models, the risk of developing PGD with donor smoking was elevated in high-risk recipients but not low-risk recipients (49). A recent study investigated donor substance use and LTx outcomes in more granularity. This group examined donor cocaine, opioid, methamphetamine, and cannabis use along with their effect on recipient outcomes post-transplantation and did not find a significant difference in development of PGD or overall survival. There was, however, an increased probability of developing PGD when the donor was a smoker (50). The temporality of smoking has also been postulated as contributing to the development of PGD and adverse outcomes. A 2020 study demonstrated acceptance of lungs from donors who were current smokers led to a higher incidence of PGD3 at 72 hours than either former smokers or never smokers (51). In addition to typical tobacco smoking, the rise of e-cigarettes and vaping is a looming public health threat with documented damage to lung tissue. A 2023 single center study found no association between donor vaping and PGD3, as well as other short term outcomes (52). However, the study only included 29 patients, and the authors cite their small sample size as a limitation of their ability to detect a difference. Given the mixed data, a nuanced approach should be undertaken when accepting lungs from a donor with prior substance use on a case-by-case basis.

### History of infection or aspiration

Assessment for airway injury, pneumonia, and aspiration is assessed during the donor operation by bronchoscopy. The ISHLT identifies aspiration events in the donor prior to explantation as a probable risk factor for PGD (53). A recent study used bile acid levels within donor bronchoalveolar lavage as a correlate for aspiration. Their data demonstrated increased rates of PGD, longer time to extubation, and shorter time to CLAD (54).

### Donor type

Donation after brain death (DBD) donors comprise the majority of pulmonary allografts in the donor pool. However, only approximately 20% of brain-dead donor lungs have been suitable for transplantation due to the many mechanisms of lung injury. In addition, brain dead donors experience a catecholamine surge following brain death that can contribute to myocardial stunning and pulmonary edema prior to donation that may contribute to the high proportion of rejected lung allografts. To expand the donor pool further, donation after circulatory death (DCD) pulmonary allografts have gained popularity over recent years (55). These comprised approximately 2% of LTx in the US in 2018, but upwards of 20% in Australia and the United Kingdom (56, 57). The lack of utilization of DCD lung donors in the United States may be attributed to concern over warm ischemia, resource waste due to uncertainty of death criteria being met upon withdrawal of support, and public scrutiny which may limit the centers that can utilize DCD donors (56). A 2018 analysis of the UNOS database was one of the first large studies examining outcomes following DCD LTx in the United States (56). There was no difference of incidence of PGD at 72 hours using DCD allografts. In addition, receipt of DCD lungs was not associated with increased hazard of death on Cox regression analysis (56, 57). Subsequent studies echoed the same sentiments of safety and feasibility (55, 57, 58). Less-than-average rates of PGD3 at 72 hours post-transplant when compared to DBD recipients (8% vs 20%) were observed as well as superior survival at both 1 and 5 years post-transplant (97% and 90% vs 90% and 61%) (57). The growing body of evidence suggests that use of DCD pulmonary allografts is safe and has acceptable incidence of PGD when compared to DBD donors. Though this has been a relatively new frontier in transplantation, this has the potential to widely expand the donor pool and decrease wait list times safely.

### Size matching of the donor allograft

Sizing of the donor allograft is important when selecting an optimal donor for a recipient. There are various methods that are employed to evaluate size matching, namely height matching, chest x ray comparison, CT volumetric analysis, and pulmonary function tests, each with their own limitations (59). This process is more complicated in Idiopathic Pulmonary Fibrosis (IPF) where lungs are often shrunken and fibrotic, requiring surgeons to estimate the size of a patient’s would-be healthy lung size. Size mismatch has been shown to correlate with negative overall outcomes and the risk of developing PGD (60). There have been several studies examining this relationship, and oversized allografts have been shown to have better overall survival and decreased incidence of PGD3 in bilateral LTx (60–64). However, oversized allografts carry their own set of challenges such as arriving to the ICU with an open chest, which itself can be a risk factor for PGD (65). A 2021 study investigated the perceived benefit of oversized allografts and found that it was not necessarily oversized lungs that conferred a benefit. Their data suggested an optimal ratio between the total lung capacity (TLC) of the donor to the TLC of the recipient between 0.8-1.2 resulted in better patient outcomes and lower rates of PGD than ratios either above or below those cutoff values (59).

A summary of donor risk factors for the development of PGD can be found in **Table 2**.

**Table 2:** Donor risk factors for PGD, ranked according to level of evidence with select corresponding references.

Donor Risk Factor	Likelihood of Contribution to PGD	References
Smoking	Definite (Current Smoker), Probable (Former Smoker)	(13, 45, 47–52)
Aspiration/Infection	Probable	(53, 54)
Alcohol Use	Probable	(46)
Size Mismatch	Probable	(59–65)
Vaping	Possible	(52)
Illicit Drugs	Possible	(50)
Donation After Cardiac Death	Unlikely	(56–58)
Donor Sex	Unlikely	(38–43)
Trauma/Pulmonary Contusions	Unlikely	(32–34)
Donor Age	Unlikely	(22–30)

## Recipient risk factors for development of PGD

### Demographics

Demographic characteristics of recipients historically implicated in the development of PGD have been thoroughly investigated and have included elevated BMI (>25, increasingly so for >30), female sex, and African American ethnicity (45, 66, 67). Recipient age has not been shown to correlate with development of PGD (45).

### Surgical history

Previous thoracic surgery has been shown to be a risk factor for adverse events postoperatively following LTx (68). Specifically, pleurodesis (either chemical or mechanical) was associated with increased postoperative morbidity and incidence of PGD; other cardiac and thoracic procedures were not independently associated with PGD development (68, 69). A more recent 2016 analysis did not identify an effect of pleurodesis on development of PGD, but only had a sample size of 10 patients and may have been too underpowered to detect a difference (70). Lung Volume Reduction Surgery (LVRS) has been considered a viable bridge to transplantation in patients with end-stage COPD. However, a study by Shigemura and colleagues demonstrated that transplantation following LVRS is technically challenging, and can impart higher perioperative morbidity and mortality, but did not confer an increased risk of PGD development (71).

### Patient conditioning

Frailty is very common in patients with end-stage pulmonary disease and is a risk factor for many perioperative complications (72). A recent study examined outcomes following LTx stratified by frailty index and found increased risk of death, but there was no increased risk of PGD in frail patients (73). Though it has not been demonstrated to be associated with PGD, frailty remains an important prognostic characteristic that portends worse outcomes following LTx.

### Diagnosis

Specific recipient diagnoses portend more risk of developing PGD postoperatively. Pulmonary hypertension, IPF, diastolic dysfunction, and sarcoidosis have been shown to be associated with development of PGD (45, 67, 74–76).

Pulmonary hypertension has historically been considered a strong prognostic indicator for the development of PGD, identified by the ISHLT consensus group in 2005 and 2016 as a risk factor (12, 53). This is likely due to the complex pathophysiology and sequelae of pulmonary hypertension in the perioperative period. Upon implantation of the donor allograft, numerous cardiopulmonary physiologic parameters change. The decreased pulmonary vascular resistance increases right ventricular output; thus, increased shear stress due to elevated compensatory cardiac output may cause injury to the underlying pulmonary vasculature. Increased right ventricular output has a cascading effect on the left ventricle; the increased volume of blood returned to the left heart may manifest as immediate diastolic dysfunction, which can potentially deliver another blow to an already sensitive pulmonary capillary bed.

Though considered a strong risk factor, the degree of impact that pulmonary hypertension has on the likelihood of developing PGD has been recently debated. A recent study found no association between pre-transplant pulmonary hypertension and PGD3 (77). However, in this single institution study, the authors only included 49 patients with a pulmonary arterial pressure >25mmHg. The small sample size and limited sample catchment area were significant limitations of this study that were identified by the authors, warranting further investigation. Recognition of the heterogeneity within patients with pulmonary hypertension may also play a role in predicting PGD development. Porteous and colleagues found that risk of PGD within this population was increased with BMI >30, female sex, degree of pulmonary artery/right atrial pressure elevation, elevated creatinine at transplant, and receipt of lungs from a smoking donor (76). Their model had high negative predictive value, but low positive predictive value. They also included both patients with primary and secondary pulmonary hypertension, which may serve as a confounder. The 2005 and 2016 ISHLT consensus statements on PGD highlight secondary pulmonary hypertension as a possible risk factor, after two studies published conflicting data on secondary pulmonary hypertension being associated with the development of PGD (12, 78).

Patients with end-stage silicosis can also be treated successfully with LTx (79). However, as this condition is quite rare, there are limited numbers of studies examining this in the literature. As patients with silicosis have been identified to have a relatively high operative risk profile due to risk of hemorrhage and significant adhesions, their overall complexity and increased time on cardiopulmonary bypass may portend more risk of developing PGD (80). The advent of robust national and international databases may give us the opportunity to examine silicosis and other pneumoconioses in more depth to examine incidence of PGD and how it differs from more common restrictive lung diseases.

Most recently, COVID-19 as well as new viral respiratory causes of ARDS have emerged as a growing public health threat. COVID-19 related interstitial lung disease has been identified in some patients after acute illness (81, 82). There have been a small number of case series published on LTx due to COVID-19 (83). In a study by Roach et al., the small sample of 183 patients with both COVID-19 ARDS and ILD collected from the UNOS database did not demonstrate any incidence of PGD (84). This was in contrast to the study by Kurihara et al. that demonstrated a 70% rate of PGD in the COVID-19 related ARDS cohort (83). A multicenter experience in LTx in other causes of ARDS demonstrated comparable short-term outcomes to those who were transplanted due to COVID-19 related ARDS (85). An issue the authors raise with transplantation in ARDS in general, is the paucity of literature evaluating short- and long-term studies outside of a handful of case reports and series. Future studies will have to be undertaken as the impact and breadth of COVID-19 related ILD evolves to better understand the impact on COVID-19 related lung disease on the development of PGD.

### Single vs double lung transplant

The most recent data suggests that transplantation of a single lung carries a two-fold increased risk of developing PGD postoperatively (45). However, this is becoming less relevant as the number of single LTx performed has stagnated and is being quickly eclipsed by the steadily growing number of bilateral lung transplants performed per year, which carry better long-term survival and less incidence of PGD.

### Anastomosis time

Anastomosis time has been implicated as another risk factor for PGD. A 2022 retrospective analysis of 427 patients examined this relationship and found a 20% increased risk of developing PGD3 per 10 minutes of anastomosis time (86). 96% of patients in this study were supported with ECMO intraoperatively, minimizing the possible impact of other forms of intraoperative cardiopulmonary support. Thus, it is critical for the transplant surgeon to operate as efficiently as possible to minimize warm ischemic time resulting from a prolonged anastomosis, in order to prevent PGD.

A summary of recipient risk factors for the development of PGD can be found in **Table 3**.

**Table 3:** Recipient risk factors for PGD, ranked according to level of evidence with select corresponding references.

Recipient Risk Factor	Likelihood of Contribution to PGD	References
Pulmonary Hypertension	Probable	(12, 53, 76–78)
African American Race	Probable	(45, 66, 67)
History of Pleurodesis	Probable	(68, 69)
Female Sex	Probable	(45, 66, 67)
Obesity	Probable	(45, 66, 67)
COVID-19 Lung Disease	Possible	(81–85)
Frailty	Unlikely	(73)
COPD	Unlikely	(45, 67)
ILD	Unlikely	(45, 67, 74–76)
Age	Unlikely	(45)

## Methods used to mitigate PGD and its effects

There are many strategies used to attenuate, mitigate, or even prevent PGD during donation, organ transportation, and during/after transplantation. Below, we will discuss some of the methods and recent pharmacotherapies in the transplant physician’s arsenal to combat PGD.

### Allograft preservation strategies/ischemic time

Organ preservation is another important aspect to consider when minimizing the risk of PGD. The gold standard of lung preservation is cold static preservation between 4-10 degrees Celsius using a low potassium solution (Perfadex) via the pulmonary artery followed by a retrograde flow in each pulmonary vein (87). Lungs are retrieved in a semi-inflated state. Cooling of the organ is provided by the perfusate solution, and direct contact of ice with the lung surface should be avoided. This method continues to be the mainstay of lung allograft transportation, although there have been numerous advances in organ preservation that are becoming increasingly used in practice.

Investigation in the 1980s demonstrated that 10 degrees Celsius was the optimal lung preservation temperature. The feasibility and implications of keeping allografts consistently at 10 degrees were not clearly understood at that time. This was partly due to the paucity of information yet to be discovered regarding cell signaling pathways and their effects on preservation (88). Since then, technology and understanding of this process have evolved. Mitochondrial injury has been implicated in limiting the extent of time organs can maintain viability. The investigators found that porcine allografts stored for up to 36 hours at 10 degrees Celsius had better pulmonary physiology when compared to lungs stored at 4 degrees Celsius. This was applied to human donors/recipients, which resulted in no incidence of PGD3 at 72 hours post-transplant, and a 100% 30-day survival for their five-patient cohort (89). This has already been implemented into clinical practice, as the LUNGguard (Paragonix) has been FDA-cleared for lung preservation, allowing precise temperature control between 4-8 degrees Celsius (90–92). Preliminary investigations have demonstrated no significant difference in rates of PGD development when compared to traditional cold storage (92). Further investigation may solidify this strategy that can not only reduce incidence of PGD, but also help to transform LTx into a semi-elective procedure. However, at this moment, the 1-year results of a clinical trial comparing this strategy to conventional cold storage has yet to be reported.

In addition to storage temperature, the duration of cold ischemic time has been implicated as a possible risk factor for PGD in previous studies (93). However, Diamond and colleagues did not find an association between ischemic time and incidence of PGD (45). Moreover, additional data echo these results, where length of cold ischemic time was not associated with development of PGD (94, 95). Interestingly, Hasenauer and colleagues reported that cold ischemic time was not significantly associated with the development of PGD in an animal model, but warm ischemic time was indeed correlated with PGD development (94). Based on the data reported, though cold ischemic time has recently been demonstrated to have no association with PGD, it is still important to minimize ischemic time, both warm and cold, to slow the metabolic rate of each allograft to minimize any free radical and accumulation of toxic metabolites that may contribute to PGD.

### *Ex-vivo* lung perfusion

Over the last decade, normothermic *ex-vivo* lung perfusion (EVLP) has been increasingly utilized to expand the donor pool, minimize cold ischemic time, and preserve donor allografts until transplantation. The INSPIRE study, a phase 3 clinical trial examining patients whose allografts were preserved using the Organ Care System (OCS) device (Transmedics) showed comparable short- and long-term survival to patients whose allografts were stored on ice (96). The authors reported significantly better rates of PGD3 relative to cold storage at 4 degrees celsius. Subsequently, the EXPAND study examined the OCS device with extended donor criteria and DCD donors (97). This study found rates of PGD3 of 44% within the initial 72 hours post-transplant, which improved to only 6% at 72 hours, had an increased proportion of donor lungs utilized, and excellent early and 1-year survival. The authors cited a large proportion of DCD allografts and transplants performed on CBP as possible explanations for the high initial rates of PGD. The OCS system, based on the EXPAND and INSPIRE trials, has had promising results, and appear to be more protective against PGD relative to static EVLP. The DEVELOP UK trial demonstrated an 88% initial PGD3 rate, and 27% of their sample had PGD3 at 72 hours for static EVLP, compared to 6% PGD3 at 72 hours observed in the EXPAND trial (97–99).

There have been several systematic reviews and meta-analyses regarding normothermic *ex-vivo* lung perfusion for allograft preservation, which did not find a significant difference in PGD3 or overall outcomes versus cold storage (100, 101). Importantly, EVLP has been performed with lungs that that would have been deemed not suitable for clinical transplantation with acceptable rates of PGD, hence these results represent an important message: EVLP to expand the donor pool safely provides more donor allografts for patients in need. Though the data is mixed regarding incidence of PGD with utilization of normothermic *ex-vivo* perfusion, short-and long-term outcomes support its use for expansion of the donor pool.

### *In-situ* thoracoabdominal normothermic regional perfusion

EVLP, as described above, is becoming a more widely used method of *ex-vivo* lung preservation. *In-situ* Thoracoabdominal Normothermic Regional Perfusion (TA-NRP) has been gaining popularity for abdominal organ preservation and has just begun to be described in small series in DCD LTx (102). The initial data on the use of TA-NRP in lung donation is mixed, extremely limited to a small number of case studies and series, which note development of PGD ranging from grades 0-3. In an 8-patient single center experience, the authors noted 7 out of 8 patients had PGD1 or less at 24 hours, and there was no observed short term pulmonary-related mortality, identifying TA-NRP as a potential strategy to increase the donor pool and protect DCD lungs from PGD (103). Further research is needed, as these studies are too small to identify an effect of TA-NRP on PGD. Of note, there is an ethical debate surrounding the use of TA-NRP. The controversy surrounds the “dead donor rule” and the argument that once circulation is restarted with TA-NRP, the patient may not be dead. The counter-argument is that TA-NRP is not started until after patients are dead, and that its initiation is not with the intent to resuscitate, rather the intent to honor their wish to donate (104). Though there is considerable ethical debate regarding this process and considerable logistical challenges with coordination between perfusionists, donor/recipient centers, and organ procurement organizations TA-NRP has the potential to expand the donor pool, and must be validated with larger, multi-center studies to better evaluate its efficacy and relationship to PGD once there is ethical consensus (105).

### Intraoperative support

The method of intra-operative mechanical circulatory support (MCS) may also play a role in protection against the development of PGD. Many patients can become hemodynamically unstable during LTx necessitating initiation of MCS. Common indications include pre-operative MCS use warranting continued use in the operative setting, hemodynamic instability immediately before transplantation, or a high risk of decompensation upon induction of anesthesia. Additionally, patients that have refractory hypoxia during transplantation (most notably after implantation of the first graft), refractory pulmonary hypertension >50mmHg (typically just before implantation, examined via occlusion of the pulmonary artery), and hemorrhage may also result in acute necessity for MCS (106). Historically, cardiopulmonary bypass (CPB) has been the method of choice used for this purpose. However, use of intra-operative cardiopulmonary bypass in LTx has been implicated in numerous studies as portending worse overall outcomes and increased risk of developing PGD (45, 67). In addition, CPB is associated with high likelihood of blood product utilization, which itself has been implicated in carrying increased risk for PGD development, especially FFP and platelets (2, 34, 107, 108). A recent study postulated that it may not be CPB itself that contributes to the development of PGD, but the amount of time spent on CPB. In an analysis of 1,039 LTx recipients, 67% of which utilized CPB at the time of their transplant, demonstrated that >3 hours of CPB is associated with severe PGD. Rates of severe PGD with <3 hours of CPB were comparable to patients who did not receive support with CPB (109).

Venoarterial Extracorporeal Membrane Oxidation (VA-ECMO) has continued to gain popularity as an alternative method of MCS and is now widely used for perioperative hemodynamic support around the time of LTx (106, 110–112). In addition, benefits of ECMO over CPB include avoidance of full heparinization which reduces the risk of hemorrhagic complications, the ability to extend ECMO into the ICU for continued circulatory support if needed, and reduction of trauma to the allograft to allow for controlled reperfusion and lung parenchyma-protecting ventilation strategies. The use of VA-ECMO routinely may help to blunt the harsh physiologic changes that occur upon implantation of the non-diseased allograft. VA-ECMO gives the transplant surgeon a high degree of control of physiologic parameters to prevent ischemia-reperfusion injury and factors contributing to PGD development.

Numerous studies continue to identify VA-ECMO as being superior to CPB in terms of risk of developing PGD postoperatively and overall outcomes (45, 113–115). A 2020 prospective study of mandatory intraoperative VA-ECMO during LTx reported excellent primary graft function with a PGD rate of 1.2% after 72 hours (116). The data are comparable with a previous retrospective study demonstrating survival benefit and low incidence of PGD postoperatively (117). The authors concluded that this strategy allowed for optimal graft handling, minimizing first-lung syndrome, improving hemodynamic stability with VA-ECMO, and reducing reperfusion injury and fluid extravasation through a controlled environment for lung implantation and reperfusion. The versatility of VA-ECMO has become a valuable tool in the LTx surgeon’s armamentarium to prevent PGD and optimize outcomes following LTx.

### Immunologic and pharmacologic strategies

Numerous induction immunosuppression agents are used in LTx, including anti-thymocyte globulin (ATG), IL2 antagonists such as basiliximab (Simulect), and alemtuzumab (Campath), an anti-CD52 monoclonal antibody (118). However, the standard of care regarding optimal induction immunosuppression has not been established, nor has a possible protective effect against PGD of varying regimens. The relationship between immunotherapy and PGD prevention has recently begun to be investigated. A retrospective single-center analysis sought to compare outcomes following induction therapy of basiliximab or alemtuzumab. Alemtuzumab was found to significantly decrease the incidence of PGD, delayed chest closure, postoperative liver dysfunction, and acute cellular rejection (ACR) within the first year as well as improved overall survival (119). Further investigation is needed to standardize immunosuppression strategies that optimize perioperative outcomes and protect against PGD.

Donor-recipient HLA status and virtual crossmatching have been very important principles in organ transplantation as matching has been demonstrated to maximize overall survival and graft function and has recently been identified as a new potential technique to prevent PGD (120–122). A recent study implicated HLA class II eplet mismatch as a negative prognostic factor in lung transplantation, associated with poor outcomes and increased risk of PGD (121, 122). As sites of eplet mismatches increase, so did the severity of PGD seen postoperatively (121). However, eplet mismatch is not routinely screened for, as it can only be detected using high-resolution HLA matching, highlighting a potential role for high-resolution HLA matching in the prevention of PGD as this technology becomes more widely available.

The use of plasmapheresis has been considered as a tool that may mitigate the development and effects of PGD. In the setting of a positive virtual cross match or in patients with donor specific antibodies (DSA) at the time of transplant, plasmapheresis has been used as desensitization treatment. Early evidence of success of desensitizing DSA+ recipients preoperatively was reported in 2011 in renal transplantation (123). A previous study examined the utility of desensitization of DSA+ recipients in LTx (124). Recipients were desensitized with plasmapheresis, intravenous immune globulin, antithymocyte globulin, and mycophenolate mofetil if they had positive pre-transplant DSA, panel reactive antibody (PRA)>30%, or medically urgent. They found no difference in PGD incidence, survival at 30 days and 1 year, FEV1, and forced vital capacity between patients who were DSA-/PRA+ and DSA+ patients who were desensitized (124). However, a 3-year prospective randomized controlled trial identified desensitization as a risk factor for developing PGD. A high percentage of PGD3 was seen in patients with high DSA levels despite desensitization. Interestingly, increased incidence of CLAD was not seen in the desensitized high DSA group which implied a protective effect on graft survival despite of the high proportion of PGD3 seen in this population (125). This field of study will play an important role in expanding the donor pool and allowing for higher access to LTx for patients who are DSA+, but the impact of desensitization on PGD must be thoroughly investigated as well (126).

There are also several recent clinical and preclinical trials investigating therapies that may mitigate ischemia-reperfusion injury and PGD following LTx. Hashimoto and colleagues observed a reduction in ischemia-reperfusion injury following LTx in a rat model with administration of a homodimer of Annexin V, which prevents cell adhesion by shielding phosphatidyl serine motifs (127). Iskender and colleagues examined the effect of intravenous alpha-1 antitrypsin (A1AT), previously shown to have a protective effect against ischemia-reperfusion injury in rat models, in a large animal model. They demonstrated decreased stigmata of ischemia-reperfusion injury and improved post-transplant lung function in a porcine model (128). Another large-animal study by LaPar demonstrated improved lung function post LTx with administration of an adenosine A2_a_ receptor agonist, a known mediator of a potent anti-inflammatory cascade (129).

The complement cascade has been previously identified as playing a role in the development of ischemia-reperfusion injury and PGD (130). Numerous anti-complement therapies have been demonstrated to show potential in reducing the extent of ischemia-reperfusion injury (131, 132).

Interleukin-10 (IL-10) has been identified previously as an anti-inflammatory cytokine, inhibiting cellular adhesion, free radical formation, and production of pro-inflammatory cytokines (133). Immunomodulation and increased expression of IL-10 has been shown in a handful of studies to be protective against ischemia-reperfusion injury (133, 134).

H19, a long-noncoding RNA, has been demonstrated to play a role in numerous pulmonary disease processes, and a 2023 study sought to examine a possible role in the development of PGD (135). They identified H19 within a signaling axis of PGD via transcriptome analysis, and when silenced in a murine model, found reduced inflammatory cell infiltration and secretion of CCL28, which itself stimulates inflammatory chemokine secretion, and decreased incidence of PGD (135). Identification of this signaling axis in PGD may allow for targeting of H19 and similar genomic pathways in the prevention of PGD. Collagen V (Col(V)) has also been identified as a possible target for immune modulation, as previous studies have implicated anti-Col(V) T cell immune activity as being involved in the development of airway injury and PGD following LTx (136). Another targeted therapy of recent interest is that of the protein kinase C (PKC) pathway. The PKC cascade has previously been associated with ischemia-reperfusion injury in the heart and brain (137). Kim and colleagues demonstrated that in LTx, knockdown of the PKC cascade via small interference RNA (siRNA) resulted in decreased expression of inflammatory mediators and changed the main mode of cell death from necrosis to apoptosis in rat models (137). There are many opportunities on the horizon to mitigate ischemia-reperfusion injury and PGD as these therapies begin to make their way from bench to bedside.

A summary of strategies/trials aimed at mitigating PGD can be summarized in **Table 4**.

**Table 4:** Strategies and clinical/preclinical trials aiming to mitigate PGD, listed in order of presentation with select corresponding references.

PGD Mitigation Strategies/Trials	References
*Ex-Vivo* Lung Perfusion	(96–101)
*In situ* Thoracoabdominal Normothermic Regional Perfusion	(102–104)
Venoarterial Extracorporeal Membrane Oxidation	(106, 110–117)
Immunosuppression	(118, 119)
High-Resolution HLA Matching	(120–122)
Plasmapheresis/Desensitization in Setting of +Donor Specific Antibodies	(124–126)
Annexin V Homodimer Therapy	(127)
Alpha-1-Antitrypsin Infusion Therapy	(128)
Adenosine A2_a_ Receptor Agonist Therapy	(129)
Anti-Complement Therapy	(133, 134)
IL-10 Immunomodulation	(133, 134)
H19 Long-Noncoding RNA Silencing	(135)
Protein Kinase C Knockdown	(137)

## Perspectives/conclusion

PGD is a major cause of morbidity and mortality following LTx. We have effectively summarized donor and recipient risk factors, as well as those pertaining to operative technique, for developing post-transplant PGD. In addition, we have compiled numerous strategies that have been used to mitigate PGD such as gene therapy, preclinical pharmacologic therapies, desensitization, and advances in mechanical circulatory support and *ex-vivo* lung perfusion. This remains a major area of opportunity for research with much potential to continue to improve outcomes following LTx.

## Author contributions

JJ: Conceptualization, Methodology, Project administration, Visualization, Writing – original draft, Writing – review & editing. RV: Supervision, Writing – review & editing. AO: Conceptualization, Supervision, Writing – review & editing. JF: Writing – review & editing. RW: Writing – review & editing. JD’C: Conceptualization, Supervision, Writing – review & editing. PR: Funding acquisition, Project administration, Resources, Supervision, Validation, Writing – review & editing, Conceptualization.
